# Metacarpal bone loss in patients with rheumatoid arthritis estimated by a new Digital X-ray Radiogrammetry method – initial results

**DOI:** 10.1186/s12891-016-1348-5

**Published:** 2017-01-06

**Authors:** Alexander Pfeil, Hans Henrik Thodberg, Diane M. Renz, Lisa Reinhardt, Peter Oelzner, Gunter Wolf, Joachim Böttcher

**Affiliations:** 1 Department of Internal Medicine III, Jena University Hospital – Friedrich Schiller University Jena, Erlanger Allee 101, 07747 Jena, Germany; 2 Visiana, Søllerødvej 57 C, DK-2840 Holte, Denmark; 3 Institute of Diagnostic and Interventional Radiology, Jena University Hospital – Friedrich Schiller University Jena, Erlanger Allee 101, 07747 Jena, Germany; 4 Institute of Diagnostic and Interventional Radiology, SRH Wald-Klinikum Gera, Straße des Friedens 122, 07548 Gera, Germany

**Keywords:** BoneXpert, Digital X-ray Radiogrammetry, Bone mineral density, Rheumatoid arthritis, Cortical bone loss, Osteoporosis

## Abstract

**Background:**

The Digital X-ray Radiogrammetry (DXR) method measures the cortical bone thickness in the shafts of the metacarpals and has demonstrated its relevance in the assessment of hand bone loss caused by rheumatoid arthritis (RA). The aim of this study was to validate a novel approach of the DXR method in comparison with the original version considering patients with RA.

**Method:**

The study includes 49 patients with verified RA. The new version is an extension of the BoneXpert method commonly used in pediatrics which has these characteristics: (1) It introduces a new technique to analyze the images which automatically validates the results for most images, and (2) it defines the measurement region relative to the ends of the metacarpals. The BoneXpert method measures the Metacarpal Index (MCI) at the metacarpal bone (II to IV). Additionally, the MCI is quantified by the DXR X-posure System.

**Results:**

The new version correctly analyzed all 49 images, and 45 were automatically validated. The standard deviation between the MCI results of the two versions was 2.9% of the mean MCI. The average Larsen score was 2.6 with a standard deviation of 1.3. The correlation of MCI to Larsen score was −0.81 in both versions, and there was no significant difference in their ability to detect erosions.

**Conclusion:**

The new DXR version (BoneXpert) validated 92% of the cases automatically, while the same good correlation to RA severity could be presented compared to the old version.

## Background

Conventional radiogrammetry measures distances in radiographs to estimate the thickness of the cortical bone partition. For over 50 years it has been used for the assessment of osteoporosis [1–3]. This method measures total width of a bone and the cortical thickness at the mid-point of the second metacarpal of the non-dominant hand. Based on the measured data the Metacarpal Index (MCI) as ratio of total bone width and cortical thickness can be calculated or the Barnett and Nordin Index as percentage cortical thickness [1]. Further investigations implement the Garn Index which characterised the cortical area [4] or the Exton-Smith index as the ratio of the cortical area to the total transverse area [5]. These indices of conventional radiogrammetry quantify the amount of cortical bone. Despite conventional radiogrammetry being relatively inexpensive and widely available, the technique has significant limitations regarding the imprecision due to the difficulty in identification of the endosteal margin and precise marking of the mid shaft location by the operator of the radiogrammetrical measurements [6].

Based on the conventional Radiogrammetry technique a digital version named as Digital X-ray Radiogrammetry (DXR) for the measurement of MCI at the metacarpal bones was developed and marketed as the X-posure System [7]. The DXR method measures MCI at the metacarpal bones II to IV with a high precision and in a highly reproducible way [8, 9]. The main field of application of the DXR is the quantification of periarticular metacarpal bone loss in patients with RA [10]. The periarticular bone loss as detected by the MCI is strongly associated with the disease activity in RA [11]. Cross-sectional studies have shown a strong association between reduced metacarpal index as measured by DXR and radiographically visible joint destruction [12–15], indicating that DXR-estimates function as surrogate marker of radiographic progression [10]. Longitudinal studies have also confirmed that early periarticular bone loss may be a predictor of subsequent radiographic joint damage [16–19].

Until now, the BoneXpert method has been used for the automatic determination of bone age in children [20–27]. A newly developed version of the BoneXpert-method is now available for the measurement of the MCI in adults. The BoneXpert-method is also able to quantify a new parameter, the Bone Health Index (BHI).

The aim of this study was to evaluate the new automated BoneXpert method in comparison with the original version of DXR in RA patients. The comparison focused on the agreement of their measurements to estimate MCI and on the correlation to the RA severity as estimated by the Larsen Score. Additionally, the potential of BHI in the quantification of periarticular bone loss was evaluated.

## Methods

### Study population

We included 49 patients (38 females and 11 males, mean (±SD) age: 66.7 years ± 5.7 years) suffering from verified RA diagnosed according to the revised criteria of the American College of Rheumatology in 1987 [28]. The median disease duration was 2.4 ± 2.5 years. The mean C-reactive protein was 11.3 mg/l and/or the mean Erythrocyte Sedimentation Rate 1st hour was 25 mm. No pre-selection regarding severity of RA or steroid therapy was performed. All patients were treated with disease-modifying antirheumatic drugs (Methotrexat *n* = 37, Leflunomide *n* = 12) (details see Table 1).

**Table 1:** Baseline characteristics

Total	*n* = 49
Women	*n* = 38
Men	*n* = 11
Age (years; mean ± SD)	66.7 ± 5.7
Disease duration (years; mean ± SD)	2.4 ± 2.5
C-reactive protein (in mg/L, mean ± SD)	11.3 ± 21.8
Erythrocyten sedimentations rate (in mm/hour, mean ± SD)	25 ± 18
Corticosteroids	
yes (mean dose 5 mg per day)	*n* = 25
no	*n* = 24
Disease modifying antirheumatic drugs	
Methotrexat	*n* = 37
Leflunomide	*n* = 12
Larsen-score (mean Score)	2.6

For all patients, radiographs of the hand were performed under standardized technical conditions (tube voltage 42 kV, exposure level 6 mAs, film focus distance 100 cm, film Agfa cruix).

### Methods

#### Scoring of hand radiographs

**Fig. 1:**
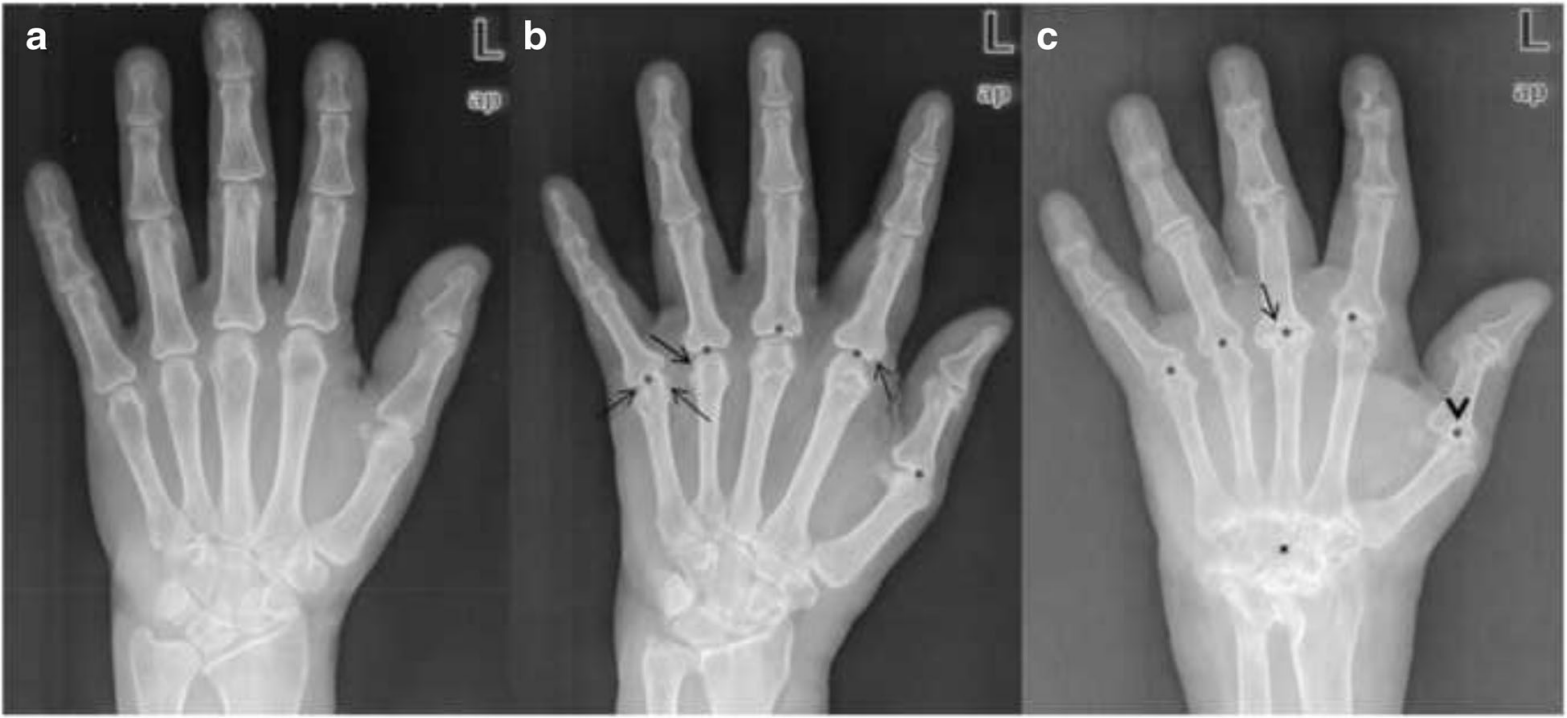
**a** Normal hand X-ray without signs of RA **b** Demineralisation of the metacarpal bone, joints space narrowing of the metacarpophalangeal joint I to V (*asterix*) and erosions of the metacarpophalangeal joint (*arrows*) **c** Severe demineralisation of the metacarpal bones as well as advanced joint space narrowing of the carpus and metacarpophalangeal joint I to V (*asterix*) with total loss of joint space at the metacarpophalangeal joint II and III, erosive destruction of the metacarpophalangeal joint III and subluxation of the metacarpophalangeal joint I

Each radiograph of the RA-cohort was scored by two radiologists blinded to each other using the modified Larsen Score which evaluates 32 joints of the feet and hands (total sum of points: 160): score 0 = normal joint; score 1 = periarticular demineralization, soft tissue affection, initial reduction of the joint space width; score 2 = initial erosions and reduction of the joint space width; score 3 = multiple erosions and advanced reduction of the joint space width; score 4 = partial ankylosis; score 5 = ankylosis or mutilation [29] (see Fig. 1). The individual sum of scoring points was then divided by the evaluated joints. In cases of ambiguity, a third highly experienced radiologist reviewed the radiographs for a final decision.

#### The X-Posure System

**Fig. 2:**
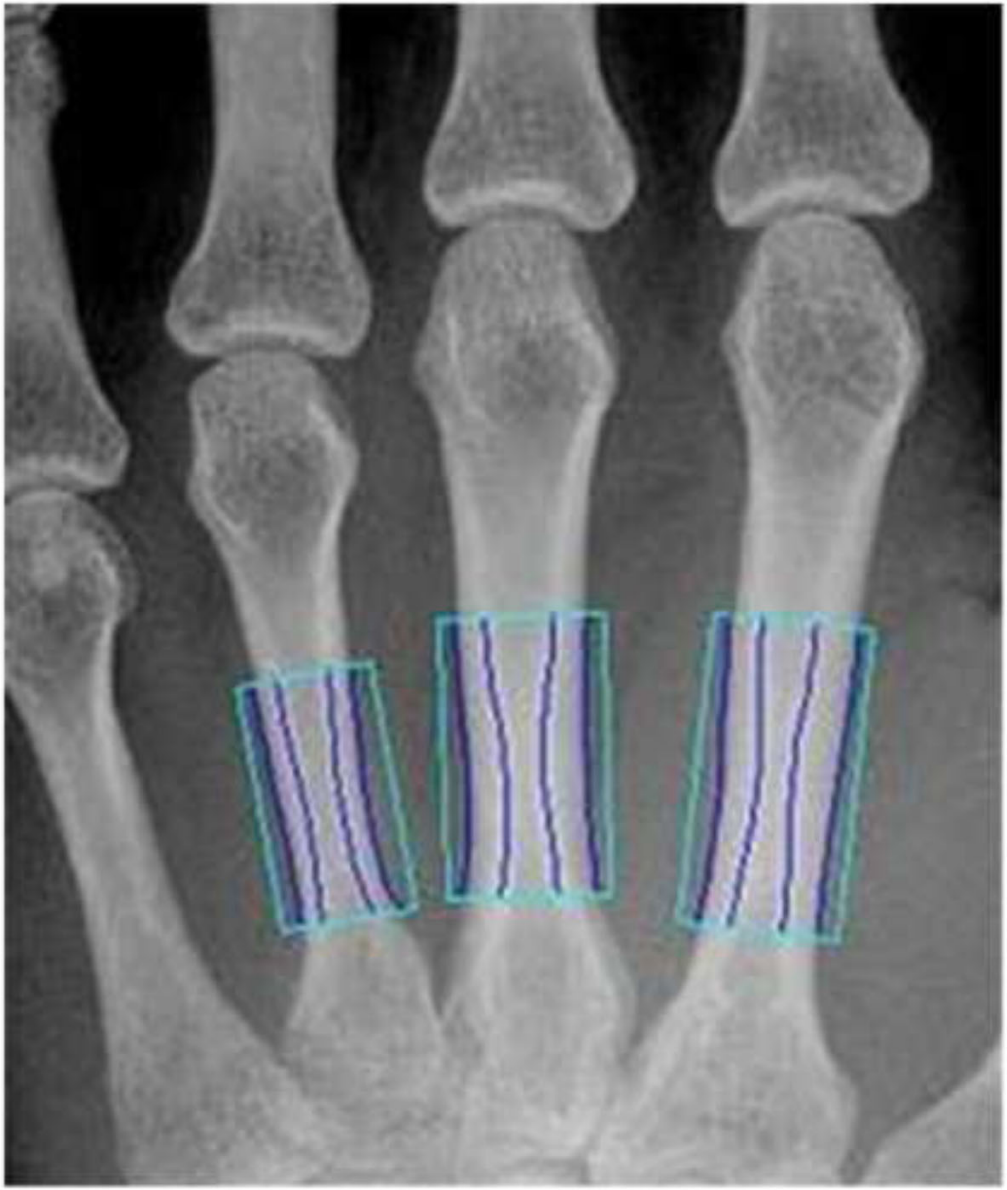
Image analysis by the X-Posure System using ROI positioning at the jointly smallest width of the metacarpal bones II to IV

The X-posure System (XP, Pronosco X-Posure System™, Version 2.0; Sectra; Linköping, Sweden, see Fig. 2) was applied to estimate cortical thickness (T_XP_), metacarpal bone width (W_XP_) and Metacarpal Index (MCI_XP_), requiring conventional or digital radiographs of the hand in an anterior-posterior projection [7].

The radiographs were digitised (Scanner UMAX Power Look 1100) in resolution 300 dots per inch by the XP-system. XP performed a continuous self-checking of the scanning process to maintain an optimal quality of the digital X-ray imaging; the analysis process is halted, if the X-ray imaging becomes inferior during the contour finding process (e. g. false identification of bone structures).

After digitalization of the hand radiographs, the method automatically defined regions of interest around the narrowest bone parts of the metacarpals II, III and IV and subsequently determined the outer and inner cortical edges of the identified cortical bone parts. There is no operator interaction in the XP-measurements. The XP-parameters were calculated as follows:

A. MCI_XP_ is a dimensionless parameter calculated as the ratio of cortical thickness to outer metacarpal width [30, 31]:
  $$ \mathrm{M}\mathrm{C}{\mathrm{I}}_{\mathrm{XP}} = 2\ {\mathrm{T}}_{\mathrm{XP}}/\ {\mathrm{W}}_{\mathrm{XP}}. $$
B. Cortical thickness T_XP_ is computed as the mean over the three bones.
C. The outer bone diameter W_XP_ was measured as the entire width of the second, third and fourth metacarpal bones including the two cortical regions and the medullary cavity. W_XP_ was calculated as the mean over the three bones [7].

#### BoneXpert adult

The BoneXpert system (BX, Version 2.1, Visiana, Holte, Denmark) is a medical device for analysis of pediatric hand X-rays for automated determination of bone age. It also includes an implementation of the DXR method for determination of bone health index in children [32]. In this study we use a new version of BX, called BX Adult (Version 2.3), which extends the DXR method to adults up to age of 90 y.

The new DXR analysis applies more advanced image analysis compared to the original implementation. Instead of locating marks only on the shafts of the metacarpals, the new method locates 32 marks all around each metacarpal II-V. This means the whole of the bones are recognised by the method and if the bones do not appear as a likely instance of such bones, it is a sign that the analysis is not reliable. In this way, the method is able to know when there is an error, in other words, it is able to validate its own recognition of the bones.

Two of the 32 marks identify the proximal and distal ends, which define the bone axis and the bone length L_BX_. A region of interest (ROI) is placed with its centre at 44% of L_BX_ from the proximal end, and the length of the ROI is 25% of L_BX_. The original method placed the ROIs at the location where the metacarpals are narrowest. We consider placing the ROIs relative the ends of the metacarpals to be anatomically more correct, and it is also more robust.

In each ROI, the outer border of the cortex is defined at the location of maximum gradient, while the inner border is at the maximum radio-opacity. The average cortical thickness T_BX_ and the bone width W_BX_ are determined in each ROI, and from these, the cortical area is defined as

$$ {\mathrm{A}}_{\mathrm{BX}}=\uppi {\mathrm{T}}_{\mathrm{BX}}{\mathrm{W}}_{\mathrm{BX}}\left(1\ \hbox{--}\ {\mathrm{T}}_{\mathrm{BX}}/{\mathrm{W}}_{\mathrm{BX}}\right). $$

Four bone indices are computed from this. Here we mention the Bone Health Index and the metacarpal index

$$ \mathrm{B}\mathrm{H}{\mathrm{I}}_{\mathrm{BX}}={\mathrm{A}}_{\mathrm{BX}}/\ \left({{\mathrm{W}}_{\mathrm{BX}}}^{1.333}{{\mathrm{L}}_{\mathrm{BX}}}^{0.333}\right) $$

$$ \mathrm{M}\mathrm{C}{\mathrm{I}}_{\mathrm{BX}}={\mathrm{A}}_{\mathrm{BX}}/\ {{\mathrm{W}}_{\mathrm{BX}}}^2 $$

These computations are done in each metacarpal, and the final indices are formed as averages over the three bones.

BoneXpert Adult validates the analysis automatically, or rejects the analysis automatically. However, a certain fraction of the images fall in an in-between category of “questionable images” where the analysis can neither be automatically accepted nor rejected. These are presented to the clinician for verification. The idea of this design is that there should as few questionable images as possible, but this feature has not previously been validated for RA patients. In this study we visually inspect all the BoneXpert analyses to verify that those automatically validated are indeed valid, and to determine the percentage of questionable images which are valid.

### Statistical analysis

The statistical analysis included the following elements

I. Evaluation of the BoneXpert self-validation.
II. For the comparison of the XP and BX methods, we define a variant of MCI for BX, because BX uses the definition MCI_BX_ = A_BX_/W_BX_^2^, while X-posure uses the definition MCI_XP_ = 2 T_XP_/W_XP_. Therefore MCI_BX-naive_ = 2 T_BX_/W_BX_ was defined . Furthermore, since the ROI is in general larger (around 30% on average) in XP than the 25% used in BX, BX finds on average larger MCI values. Therefore, a correction factor is defined to adjust for this, and an adjusted MCI is defined as MCI_BX-adj_ = MCI_BX-naive_/1.084, where the factor was derived from the data in this study.
III. The relation of XP- and BX-parameters to Larsen-Score 1 and 5 is assessed by the correlation, and we test for significant difference in correlation by a boot-strapping method, i.e. using resampling of the data with replacement, reference: Armitage page 298 an onwards, https://archive.org/details/StatisticalMethodsInMedicalResearch.
IV. The relation of XP- and BX-parameters to occurrence of erosion is assessed by the correlation, and again we test for significant differences by a boot-strapping method.

The significance level was set to *p* < 0.05.

## Results

### Self-validation of the BoneXpert

**Fig. 3:**
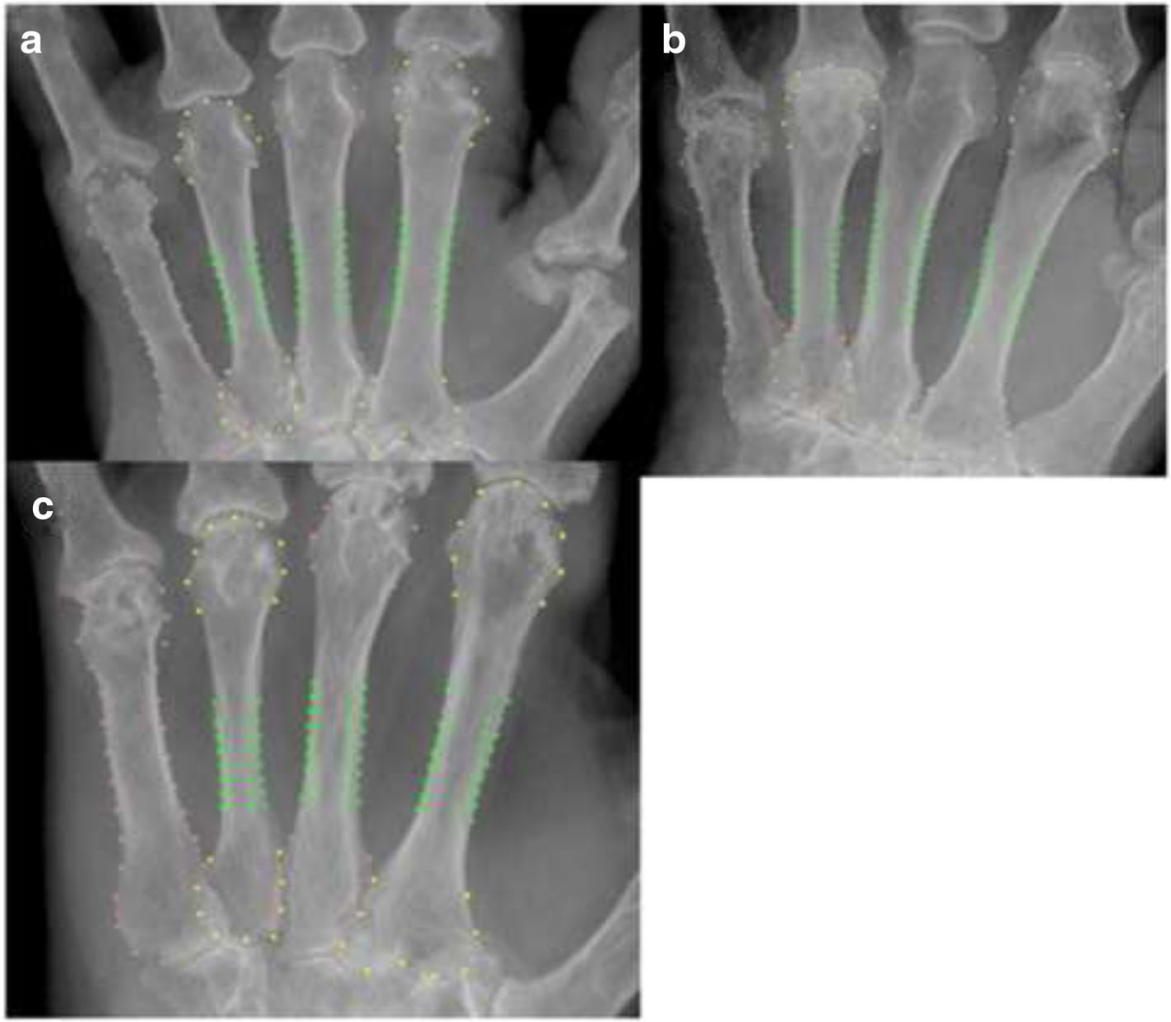
Examples of BoneXpert analyses of three images with relatively advanced RA showing how the borders of metacarpals have been located. These three cases were “self-validated” by BoneXpert. **a** strong erosion at caput 5, **b** Strong erosion at caput 5 and a slight segmentation error at caput 2 and 4, **c** Strong erosions at all four caputs, but despite this, a rater accurate segmentation of the metacarpal bones

BX was able to analyse all 49 images, and 45 were automatically validated. The 4 images flagged for visual clearance were all found to be correctly analysed by BX. Three of these “questionable” radiographs had Larsen Score 5 and one had Larsen Score 4. The XP method requires visual clearance of all images, and all were found to be correctly analysed. So both methods were able to analyse cases with extensive joint involvement caused by RA. Some examples are shown in Fig. 3.

### Comparison of the Metacarpal Index between both techniques

**Fig. 4:**
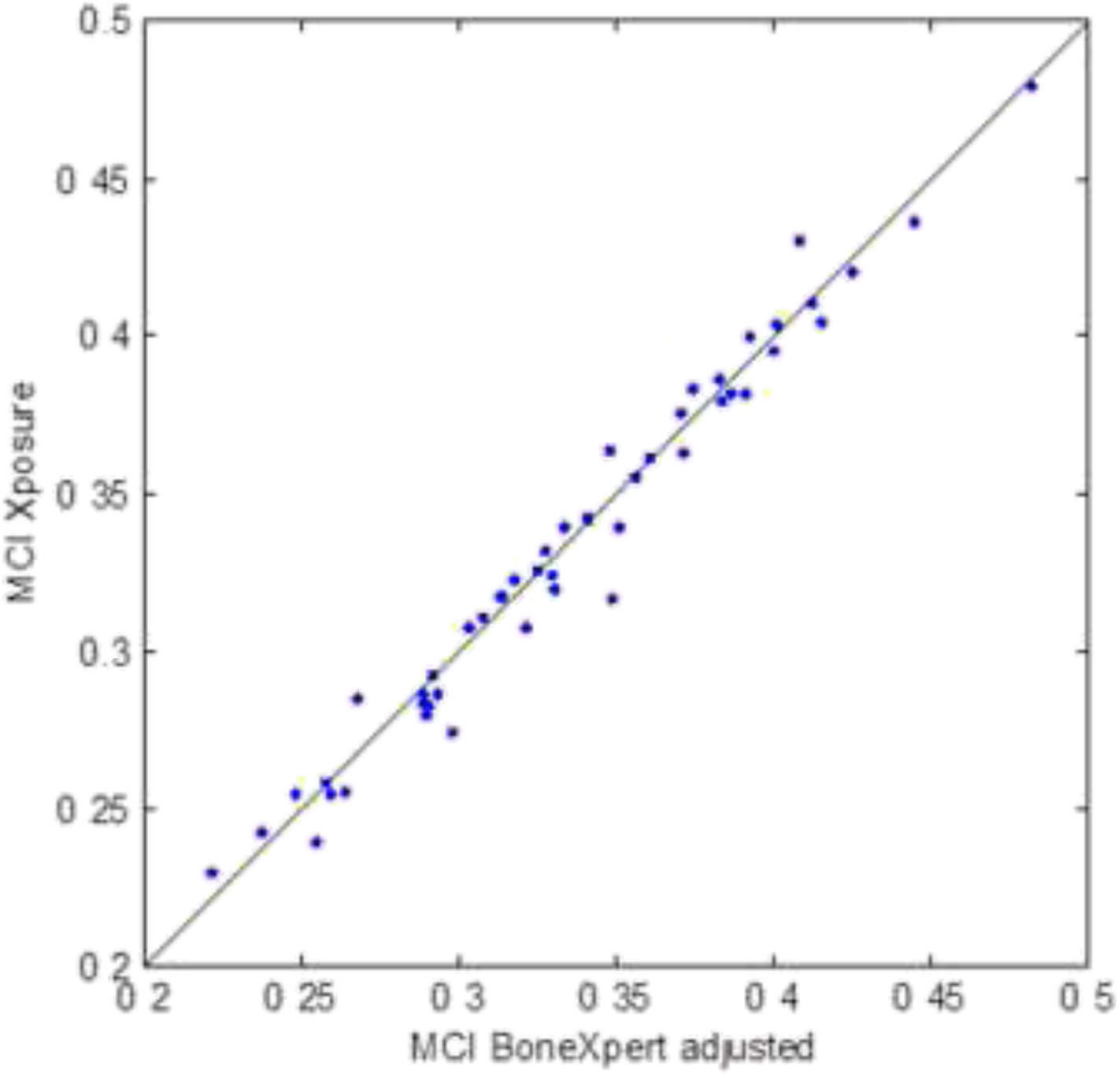
Comparison of MCI determined by the two methods, showing a very good correlation (*r* = 0.987)

The comparison of XP and BoneXpert MCI is shown in Fig. 4. It reveals a good agreement: The SD between them is 2.9% of the mean MCI. The coefficient of correlation between MCI_XP_ and MCI_BX-naive_ was *r* = 0.987 (*p* < 0.001). Comparable results were documented for T_XP_ and T_BX_ (*r* = 0.991, *p* < 0.01).

### The association between Metacarpal Index and severity of rheumatoid arthritis

**Fig. 5:**
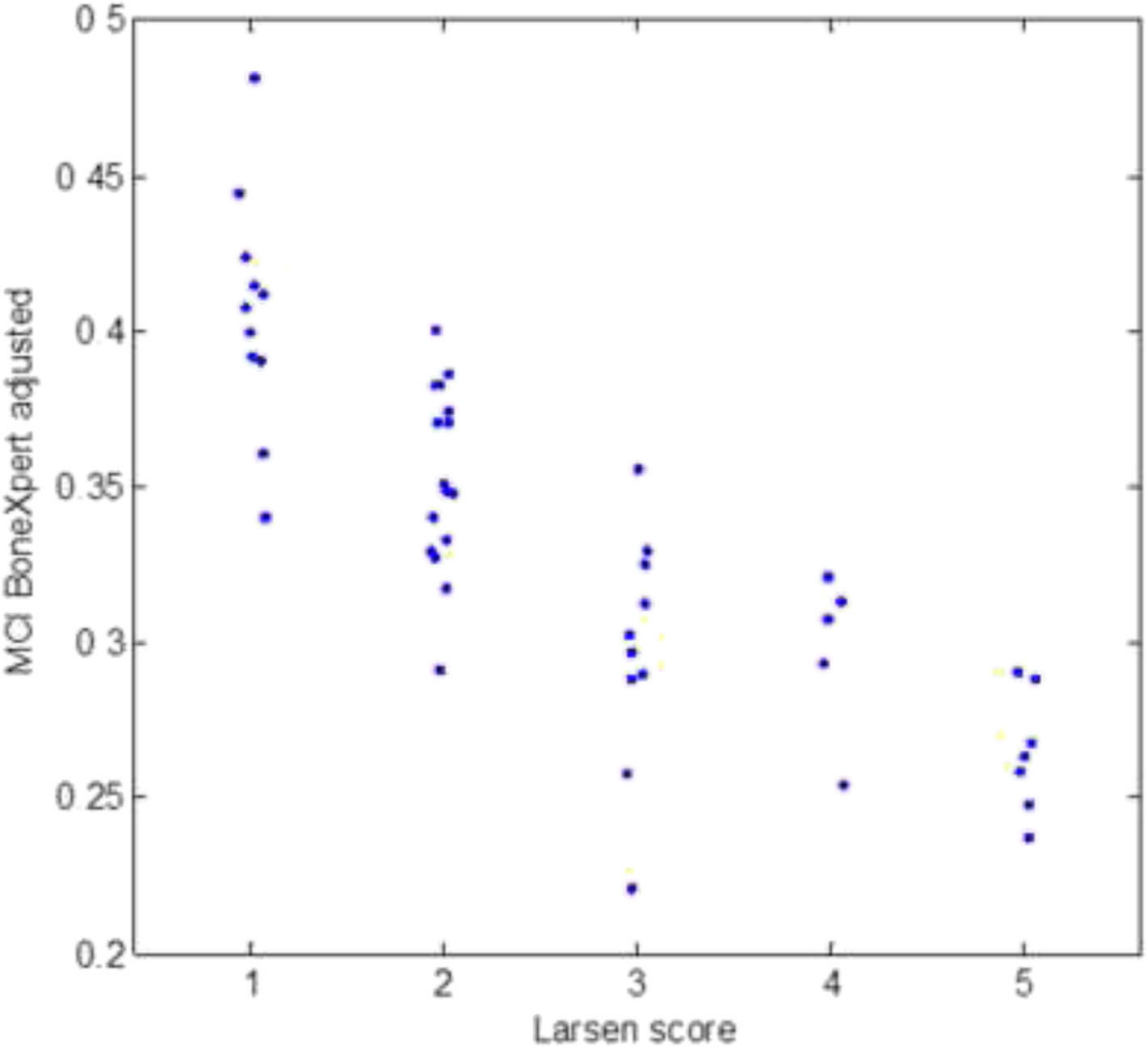
Comparison of MCI_BX-adj_ and the Larsen Score

The relation between MCI determined by BX and the Larsen Score is shown in Fig. 5. The coefficient of correlation is *r* = −0.811 for BX. It is *r* = −0.807 for the X-posure system. The bootstrapping method showed that this difference is not statistically significant – it would need to be at least 0.026 to be significant; here it is 0.004.

### The association between Metacarpal Index and erosions

**Fig. 6:**
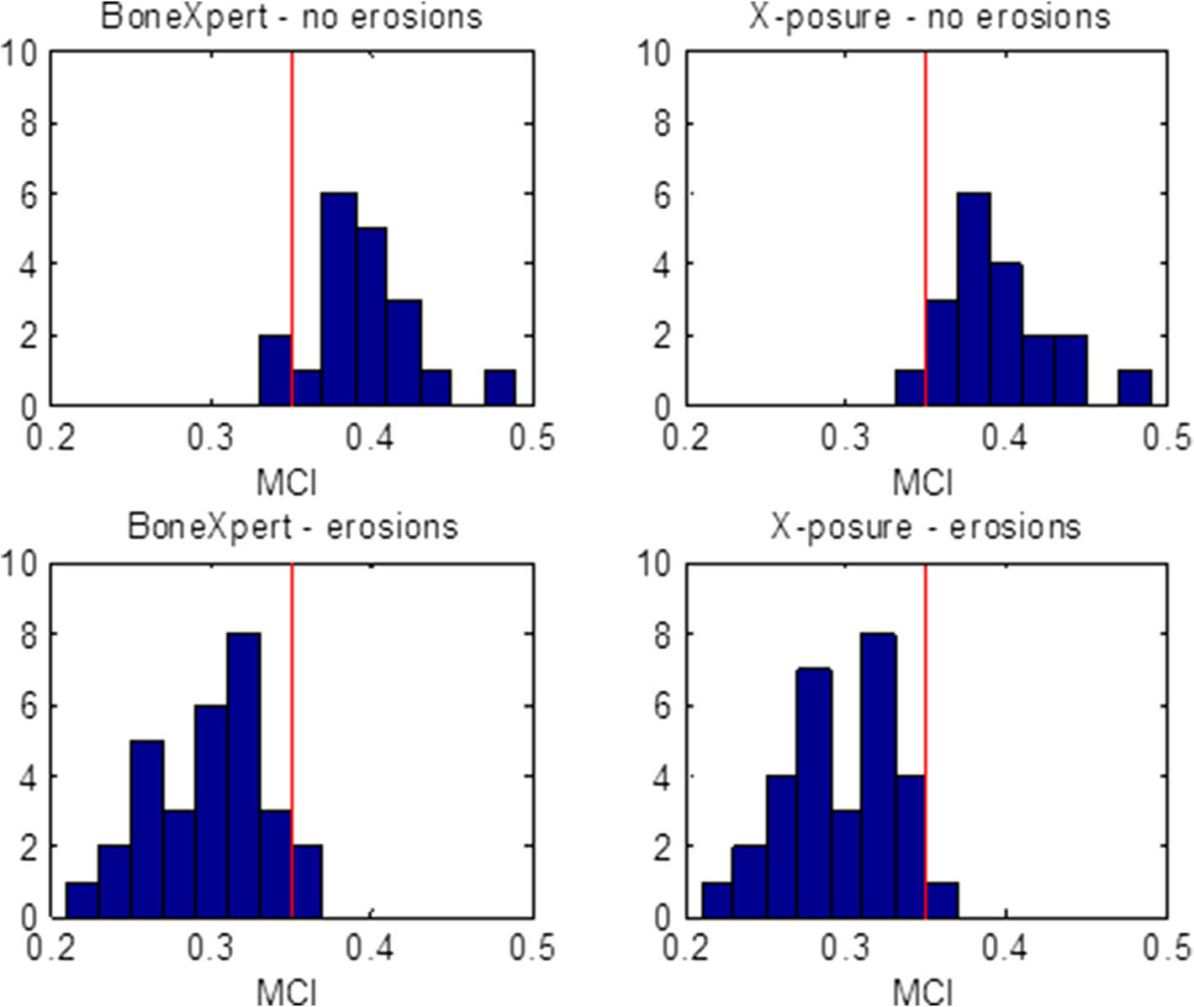
Histograms of Metacarpal Index as determined by BoneXpert (MCI_BX- adj_) and X-posure System (MCI_XP_) for patients with and without erosions. The read lines indicate a fixed threshold for separating the patient group with erosions showing a small fraction of misclassifications

Figure 6 shows histograms of MCI for cases with and without erosions.

To test whether one method is better than the other in separating these two groups, we computed the correlation between MCI and a 0–1 variable indicating erosions. The correlations were *r* = −0.828 for the X-posure system and −0.807 for BoneXpert. The bootstrapping method showed than this difference is not significant.

Furthermore, the patients with erosions presented a significantly reduced MCI for both techniques (MCI_XP_: −24.1%; MCI_BX-naive_: −19.2%).

### The Bone Health Index for the quantification of periarticular bone loss in rheumatoid Arthritis

The BHI presented a negative coefficient of correlation (*r* = 0.595, *p* < 0.01) to the Larsen Score. In this context, a reduction of BHI between the Larsen Score 1 to Larsen Score 5 was verified with 29.5% (*p* < 0.05). RA-patients with erosions revealed a significant lower BHI with −24.0% compared to the RA-patients without erosions.

## Discussion

The BoneXpert-technique is a new developed automated Digital X-ray Radiogrammetry method for the measurement of the Metacarpal Index. The objective of this study was the comparison of the BoneXpert technique to the established X-posure system considering the agreement of their measurements of the MCI as well as their correlation to the Larsen score and erosions.

### Correct placement of ROIs

The RA-related destructions of the metacarpal bone, especially at the metacarpal joints [33] could present a challenge for a method like BoneXpert which relies on locating the metacarpal ends.

Likewise, the X-posure System could place the ROIs on the wrong bones or obviously wrong (too distal or too proximal), perhaps due to artefacts of the radiographs or abnormal bone shapes.

The new BoneXpert method includes a method for self-validation of the analysis. In this study 45 hand radiographs of the patients with RA and with different stages of radiographic destruction were automatically accepted by the self-validation process of the BoneXpert technique. The remaining 4 cases required visual verification and were found to be analysed correctly. These results show that BX works reliably in this patient group. XP was also found to work reliable on all images.

A potential advantage of the new BX method is its ability to self-validate the image analysis, which could lead to a better workflow and a more reliable result. More studies are required to demonstrate such an advantage, for instance including images of poorer quality which challenge the methods more. In this study all images were analysed correctly by both methods, and this is suspected not to be true in general.

A potential advantage of the new BX method is its more appropriate definition of measurement ROI, which adjust its size to the patient size. It also allows the method to be used consistently on images with unknown magnification. However, the comparison with RA severity in this study showed no advantage of BX. Larger studies are required to clarify this issue.

### Comparison of Metacarpal Index estimated by the X-posure System and the BoneXpert

A direct comparison of the absolute MCI values is not possible, based on different procedures of the ROI positioning and configuration by the BX- and XP-techniques. But the study revealed a high correlation (*r* = 0.983, *r* ^2^ = 0.966, *p* < 0.001) of the MCI between the X-posure System and the BoneXpert. The XP-System centres the ROI at the narrowest point of the metacarpal shaft of the metacarpal bones II to IV. And it used a fixed size of the ROI. The BX-technique centres the ROI 44% from the proximal end of the metacarpal bone and the ROI scaled 25% to the bone length. The ROI location of the BX allows a more anatomically correct location of the metacarpal bone.

### Quantification of Metacarpal Index

Using the Larsen Score, MCI_BX_ (*r* = −0.811) and MCI_XP_ (*r* = −0.807) presented a significant correlation to the severity of RA. A study of Böttcher et al. (2006) also revealed a high correlation with different radiographic scoring methods in RA [34]. In this context, the MCI as surrogate marker for the periarticular demineralization could be reliably quantified by XP and BX. Periarticular demineralization of the metacarpals has been implemented as a diagnostic feature to classify bone involvement in RA, both in the Steinbroker Score as well as by the Larsen Score [35, 36]. Periarticular loss at the metacarpal bones has obviously functioned as the first radiological sign of RA considered by the scoring methods, and may be found before erosions or joint space narrowing occurs. Additionally, different cross-sectional studies have observed a strong relationship between reduced periarticular cortical bone mass as measured by DXR and radiographic joint destruction [12, 17, 34].

### The relation of erosions and Metacarpal Index

We also explored BX and XP for their diagnostic impact in the assessment of erosions. Our data revealed a significant reduced BX-MCI and XP-MCI in RA-patients with erosions. These results clearly demonstrate that periarticular osteoporosis as quantified by MCI is a predictive value in the detection of erosions.

### The Bone Health Index as a new parameter of the quantification of periarticular demineralization

We have presented data for two indices of cortical bone: MCI and BHI. They differ by using different ways to adjust for the size of the patient. The MCI is dimensionless, so it so to speak makes a complete compensation for the size, and it has the advantage that its measurement is invariant to a magnification of the X-ray image. The BHI makes a different adjustment to the dimensions of the patient, including the bone width as well as the bone length. When the BHI was introduced [32] it was conjectured that its particular adjustment was clinically more relevant than the one used in other indices, including the MCI, but it remains a hypothesis. In the data we have presented here, there is no significant difference in the performance or clinical relevance of BHI and MCI.

In this context, periarticular demineralisation, which is a characteristic feature of inflammatory bone involvement in RA, may be more valuable in the early diagnosis of RA as joint space narrowing and erosions. As demineralisation is difficult to ascertain by simple visualization of radiographs [36], BX and XP offer the benefit of a reliable quantification of periarticular bone mass in an observer-independent and highly reproducible manner [8].

One limitation of the study is based on the size of the study cohort, which reflected the proof of concept of the study. In this context, longitudinal studies were necessary to evaluate the diagnostic performance of the BX-technique, especially under consideration of therapeutic regimes in RA.

## Conclusion

In conclusion, the development of Digital X-ray Radiogrammetry has promoted the precise quantification of Metacarpal Index [37] and hand bone mass calculated by BX and XP. The clinical use of the BX-technology also allows the measurement of MCI as the traditional XP-system in patients suffering from RA. Consequently, BX-MCI is able to function as a characteristic surrogate marker for RA progression which also may improve the planning of appropriate individual therapeutic strategies.

## Abbreviations

A_BX_: Cortical area in mm^2^ as estimated by BoneXpert
BHI: Bone Health Index estimated by BoneXpert
BX: BoneXpert method
DXR: Digital X-ray Radiogrammetry
L_BX_: Length of the metacarpal bone in mm as measured by BoneXpert
MCI_BX_: Metacarpal Index as measured by BoneXpert
MCI_BX-adj_: MCI_BX-naïve_ / 1.084
MCI_BX-naïve_: Metacarpal Index as measured by BoneXpert with a definition similar to the one used in X-posure
MCI_XP_: Metacarpal Index as measured by X-Posure System
RA: Rheumatoid arthritis
ROI: Region of interest
T_B_: Cortical thickness of the metacarpal bone in mm as measured by BoneXpert
T_XP_: Cortical thickness of the metacarpal bone in cm as measured by X-Posure System
W_BX_: Average width of the metacarpal bone in mm as measured by BoneXpert
W_XP_: Average width of the metacarpal bone in cm as measured by X-Posure System
XP: X-posure System
